# Turkish Healthcare Workers’ Personal and Parental Attitudes to COVID-19 Vaccination From a Role Modeling Perspective

**DOI:** 10.7759/cureus.22555

**Published:** 2022-02-24

**Authors:** Selim Öncel, Müge Alvur, Özlem Çakıcı

**Affiliations:** 1 Division of Pediatric Infectious Diseases, Department of Pediatrics and Child Health, Section of Internal Medical Sciences, Faculty of Medicine, Kocaeli University, Izmit, TUR; 2 Department of Family Medicine, Section of Internal Medical Sciences, Faculty of Medicine, Kocaeli University, Izmit, TUR

**Keywords:** turkey, sars-cov-2, health personnel, covid-19 vaccines, child, anti-vaccination movement

## Abstract

Introduction

As in many other countries, healthcare workers (HCWs) have been identified as the priority group for vaccination in Turkey for they are in close contact with not only patients with COVID-19 to whom they provide treatment but also asymptomatic individuals with COVID-19 infection while inoculating COVID-19 vaccines. As a result of this prioritization, they will always be in the limelight and regarded as role models for personal and parental acceptance of COVID-19 vaccines.

Methods

Turkish healthcare workers (n=1,808) were contacted and invited to fill out an online questionnaire between December 27, 2020, and January 14, 2021, in order to reveal their approaches to COVID-19 vaccines and vaccination.

Results

Most of the participants had moderate concerns of having severe COVID-19. Anxiety on the adverse effects of COVID-19 vaccines was more prevalent in females and among 36- to 50-year-old healthcare workers and less frequent in physicians, nurses, and midwives and in those with a higher level of knowledge about COVID-19 vaccines. Strict anti-vaccination tendency was higher in professional categories other than physicians, nurses, and midwives. Females, physicians, nurses, midwives, healthcare workers aged 51 and over, healthcare workers having children, married healthcare workers, and healthcare workers who use scientific journals and World Health Organization (WHO) announcements as sources of information were more inclined to accept COVID-19 vaccines. The elimination of COVID-19 vaccine hesitancy in healthcare workers would be possible if people around them, physicians, and ministers or high officials get vaccinated but will persist in 19% of the healthcare workers. More than half of the healthcare workers thought vaccination against COVID-19 should not be mandatory. The Pfizer-BioNTech vaccine was the most preferred COVID-19 vaccine (37.3%). The reasons for this preference were the trustworthiness of the country of origin, the manufacturer company, the Turkish origin of its developers, the vaccine’s being the first to receive emergency validation, and its non-Indian, non-Russian, and non-Chinese origin. Parental vaccine refusal and hesitancy were present in 15.6% and 31.9% of the healthcare workers, respectively. The mistrust in COVID-19 vaccines among Turkish healthcare workers was directed toward not only pharmaceutical companies but also health authorities and academicians because of their unconvincing, conflicting, or vague statements and toward certain countries known for their production of low-quality merchandise in the past.

Conclusion

The parental COVID-19 vaccine hesitancy of 32% of the healthcare workers is unacceptably high for role modeling against anti-vaccine movement and should be diminished by implementing necessary measures as soon as possible.

## Introduction

Vaccines against coronavirus disease 2019 (COVID-19) currently seem to be the only scientifically plausible remedy for getting this deadly illness under control. In the current situation in Turkey, in which the highly contagious Omicron variant, whose *R*_0_ could be as high as 10, tends to predominate (currently 84%) over the Delta variant, with an *R*_0_ of just under 7, the average *R*_0_ may have reached 9.5, which means 90% of the population should be immune to SARS-CoV-2 [1]. Just one year ago, vaccination in children and adolescents was not considered in the short run because of a lack of safety and efficacy data in this age group. Since the pediatric population (0-17 age group according to the definition of the United Nations) constituted 27.5% of Turkey’s population, almost all adults in Turkey would then have to be vaccinated before children in order to achieve the herd immunity threshold [2,3]. As a result, there would be zero tolerance for vaccine hesitancy for the adult population, including delays or refusals in immunization processes, which stood as the greatest obstacle in front of the progress against COVID-19 control. Then, pediatric COVID-19 vaccination began for both the completeness of public immunization and the observation that children were affected frequently and severely enough by COVID-19, such as with the emergence of new SARS-CoV-2 variants, increasing the number of pediatric patients and subsequent surging of cases of multisystem inflammatory syndrome in children (MIS-C). The vaccination of the Turkish pediatric population of 12 years of age and over started on September 5, 2021, with two COVID-19 vaccines currently administered to adults: CoronaVac (generic name: COVID-19 vaccine (vero cell), inactivated) and Pfizer-BioNTech COVID-19 (generic name: COVID-19 vaccine, Pfizer) [4]. This time, the government was faced with the problem of parental vaccine hesitancy or rejection in addition to personal vaccine refusal.

According to the World Health Organization (WHO), healthcare workers (HCWs) are all people engaged in the promotion, protection, or improvement of the health of the population [5]⁠. This definition encompasses both direct HCWs, such as physicians and nurses, and indirect HCWs, such as aides, helpers, laboratory technicians, and even medical waste handlers. There are approximately 59 million HCWs worldwide, 1,061,635 of which reside and work in Turkey. Physicians and nurses constitute 16% and 19% of this grand army of workforce, respectively [6]⁠.

As in many other countries, HCWs have been identified as the priority group for vaccination in Turkey for they are in close contact with not only patients with COVID-19 to whom they provide treatment but also apparently healthy individuals while inoculating COVID-19 vaccines. As a result of this prioritization, they will always be in the limelight and inevitably regarded as role models for personal and parental acceptance for COVID-19 vaccines. Therefore, their intentions to be and get their children vaccinated are key determinants for a successful national COVID-19 vaccination program.

## Materials and methods

Study design and sample size

In this survey study, healthcare workers meeting the WHO definition mentioned above (n=1,808) were contacted and invited to fill out an online questionnaire on the platform Google Forms between December 27, 2020, and January 14, 2021, through the institutional electronic messaging system in a state university hospital by means of e-mail in two private hospitals and in the authors’ social network groups. The sample size was calculated as 385 with a confidence level of 95%, a margin of error of 5%, and population proportion of 50%.

Survey design

Healthcare workers were asked to complete a pretested online questionnaire consisting of a Likert scale, answer text type, and multiple-choice questions. The participants were divided into age groups as follows: 18-35 years, 36-50 years, and 51 years and over.

Healthcare workers were evaluated under six professional categories: physicians, nurses, midwives, medical technicians, aides or helpers, and others.

Statistical analysis

Descriptive statistics were given as absolute and relative frequencies (%). Statistical analyses between quantitative and categorical variables were done using Pearson’s chi-square test, Kolmogorov-Smirnov test, and Kruskal-Wallis one-way ANOVA, where appropriate. Statistical significance was regarded as *P*<0.05. All statistical analyses were done using TURCOSA cloud-based software by Turcosa Analytics (www.turcosa.com.tr).

Data availability

The data that support the findings of this study are deposited on Mendeley Data and Turcosa Analytics website (www.turcosa.com.tr). They will be made available to bona fide researchers by the corresponding author upon reasonable request and subsequent agreement by the authors.

Ethical considerations

This study was carried out by means of the ethical approval granted by the Kocaeli University Noninterventional Clinical Research Ethics Committee (reference: GOKAEK-2020/22.13) and the written permission from the Ministry of Health.

## Results

Study population

Of the 1,808 participants, 1,801 (1,227 (68.1%) females and 574 (31.9%) males) HCWs who chose to mention their professional category consisted of 927 (51.5%) physicians, 396 (22%) nurses, 83 (4.6%) midwives, 80 (4.4%) medical technicians, 93 (5.2%) aides or helpers, and 222 (12.3%) others. The participants were mostly 18-35 years of age (n=780) (43.3%), followed by those aged 36-50 years (n=664) (36.9%), and those 50 years and over (n=357) (19.8%). The majority (n=1,278) (70.9%) of the participants were married. The HCWs who have recovered from COVID-19 constitute 17.9% (n=323) of the participants. A similar proportion (17.8%) (n=320) of HCWs had at least one individual in their household. Three hundred forty-two (19%) HCWs had a medical condition that placed them at increased risk of having severe COVID-19. Overall, 110 (6.1%) HCWs had volunteered to take part in a COVID-19 vaccine study.

Risk perception of having severe COVID-19

When asked about their risk perception of having severe COVID-19 on a scale of 1-5, with “1” being very low and “5” being very high, most HCWs (n=744) (41.4%) expressed their concern as “3” (moderate). The risk perception of nurses was significantly higher than that of aides and helpers, medical technicians, and other HCWs, excluding physicians and midwives (*P*=0.005, *P*=0.005, and *P*<0.001, respectively).

Being knowledgeable about COVID-19 vaccines

Of the HCWs, 83% declared that they were knowledgeable about COVID-19 vaccines, whereas 847 (47.2%) had doubts about the sufficiency of their knowledge. Being knowledgeable was associated with increasing age (*P*<0.001).

Sources of information

The sources from which this knowledge was obtained were newscasts (n=1,092) (60.8%), social media (n=1,152) (64.2%), scientific journals (n=1,140) (63.5%), and announcements by the Ministry of Health (n=1,412) (78.5%) and WHO (71.4%).

Perceptions of short-term and long-term side effects of COVID-19 vaccines

**Figure 1:**
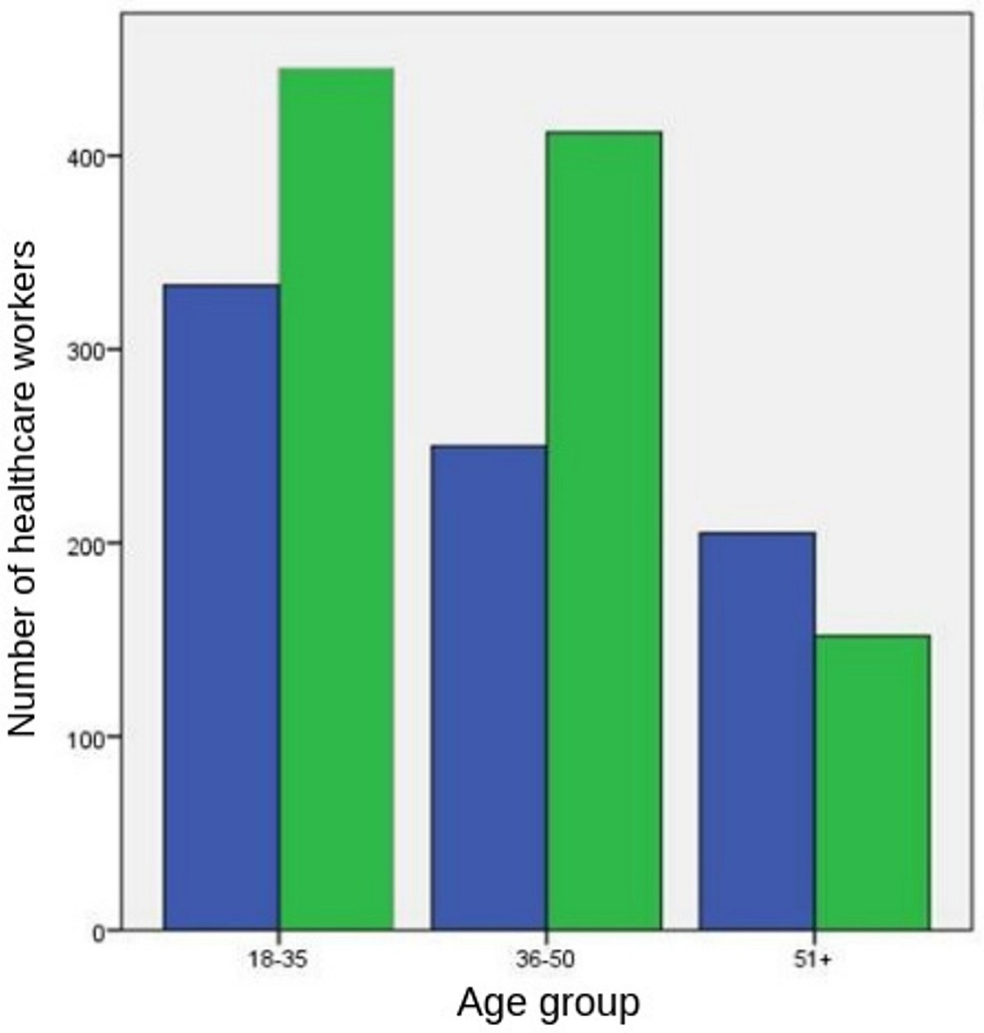
Healthcare workers’ reported anxiety on the short-term adverse effects of COVID-19 vaccines The left and right columns in each pair refer to healthcare workers not reporting and reporting anxiety, respectively.

**Figure 2:**
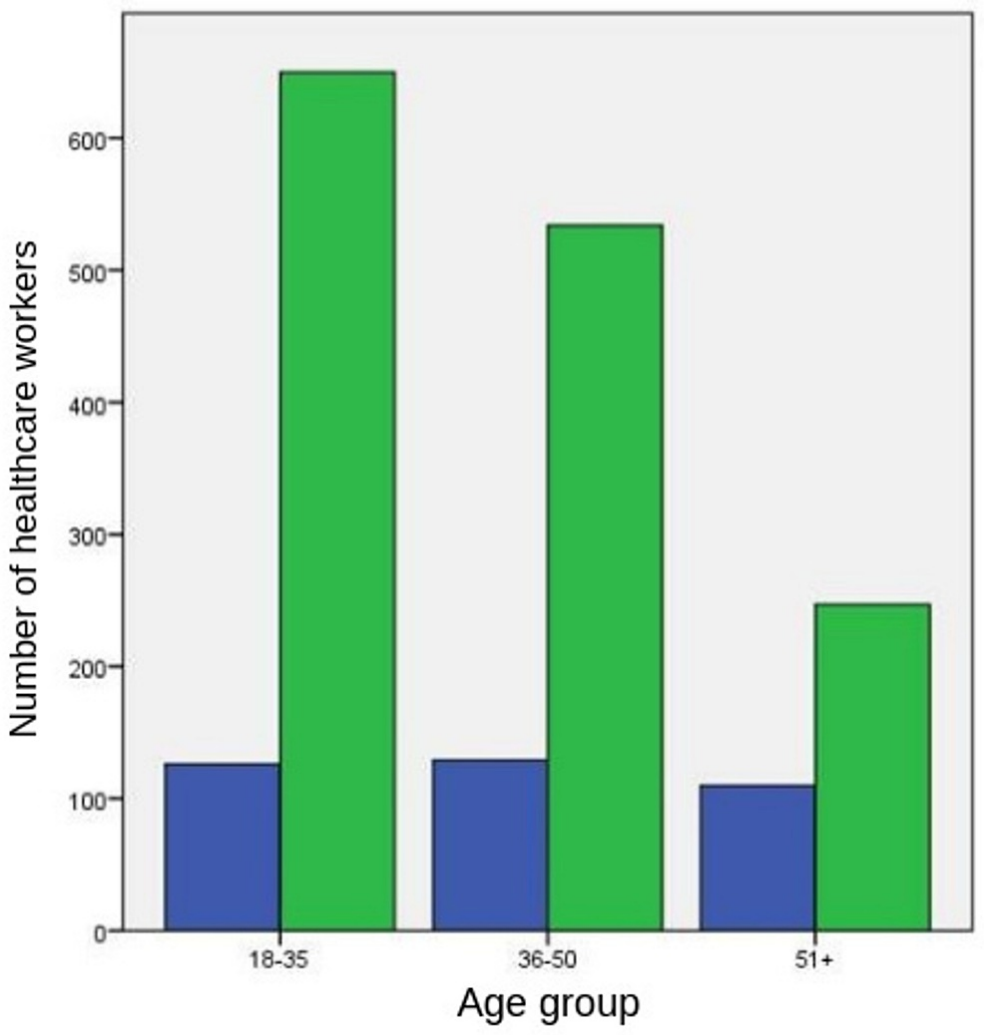
Healthcare workers’ reported anxiety on the long-term adverse effects of COVID-19 vaccines The left and right columns in each pair refer to healthcare workers not reporting and reporting anxiety, respectively.

The probable short-term and long-term adverse effects of COVID-19 vaccines created anxiety on 1,010 (56.2%) and 1,433 (79.7%) HCWs, respectively. Anxiety on short-term and long-term adverse effects was statistically more prevalent in females (83.3% versus 61.2%) and among 36- to 50-year-old HCWs than their elder counterparts (80.5% versus 69.2%) (*P*<0.001), which was inversely associated with age (*P*<0.001 for both) (Figures 1, 2).

Anxiety levels were lower in both short and long term in physicians, and nurses and midwives combined compared to other professional categories (medical technicians, aides or helpers, and others combined) (51.5% and 78.3%, respectively, versus 72.8% and 84.5%, respectively) (*P*<0.001 and *P*=0.004, respectively). The presence of anxiety about the adverse effects of COVID-19 vaccines in both short and long term decreased with knowledgeableness about COVID-19 vaccines.

The proportions of HCWs who were knowledgeable and anxious about the short-term adverse effects of COVID-19 vaccines and those who were not knowledgeable but anxious about the short-term adverse effects of COVID-19 vaccines were 51.4% and 79.2%, respectively (*P*<0.001). The proportions of HCWs who were knowledgeable and anxious about the long-term adverse effects of COVID-19 vaccines and those who were not knowledgeable but anxious about the long-term adverse effects of COVID-19 vaccines were 77.2% and 91.9%, respectively (*P*=0.004).

Strict anti-vaccination tendency

Seventy-four (4.1%) HCWs were against all vaccines including COVID-19 vaccines. No difference was observed between the female and male strict anti-vaxxers (n=50 (4.1%) and n=24 (4.2%), respectively) (*P*=0.51). However, among those who do not have any children and in professional categories other than physicians, nurses, midwives, and nurses as a whole, the anti-vaxxer tendency was significantly higher than in the rest of the HCWs (6% versus 3.1% (*P*=0.006) and 9.7% versus 2.6% (*P*<0.001) within the mentioned groupings, respectively).

Opinion on the efficiency of COVID-19 vaccines

Of the HCWs, 28% (n=509) thought that COVID-19 vaccines would not be efficient at all. This pessimism was significantly associated with un-knowledgeableness about COVID-19 vaccines (23.7% versus 50.2%) (*P*<0.001).

Acceptance of COVID-19 vaccines

Over one quarter (n=482) (26.8%) of the HCWs said that they would refuse to be immunized with the COVID-19 vaccine offered by the government. There was a significant difference in the prevalence of COVID-19 vaccine refusal in terms of gender (n=355 (29.1%) among females versus n=127 (22.2%) among males) (*P*=0.002). The group of eldest HCWs (those 51 years and over) had the highest willingness for COVID-19 vaccines (n=308) (86.3%).

**Figure 3:**
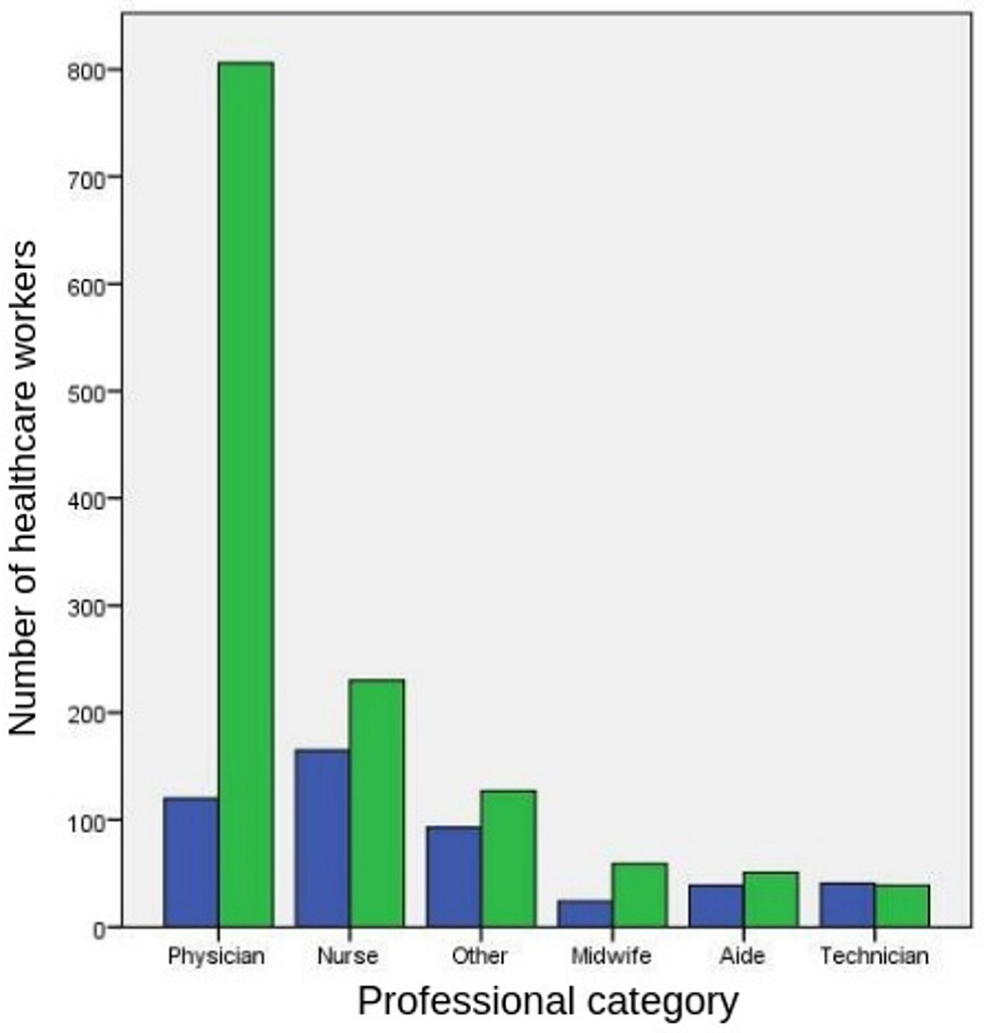
Acceptance of COVID-19 vaccines among the professional categories of HCWs The left and right columns in each pair refer to HCWs refusing and accepting COVID-19 vaccines, respectively.

Of the 1,794 responding physicians, nurses, and midwives, 73% were willing to get vaccinated. The willingness among the medical technicians, aides and helpers, and others were much lower (55.6%) (*P*<0.001). The acceptance of COVID-19 vaccines among professional categories of HCWs is shown in Figure 3.

Having children was a factor significantly in favor of COVID-19 vaccine acceptance such that the number of HCWs who were willing to be immunized against SARS-CoV-2 infection was 909 (77.1%) in 1,179 respondents with children. It was much less (n=402 (65.5%)) in 615 respondents having no children (*P*<0.001).

The acceptance of COVID-19 vaccines among HCWs having a chronic illness (n=342) was higher than those who do not. However, it was not significant (n=263 (76.9%) versus n=1,050 (72.3%) (*P*=0.09).

Vaccine acceptance did not differ significantly between HCWs living with a person with a chronic illness (n=545) (74.9%) and HCWs living in a household consisting of individuals without a chronic illness or HCWs living alone (n=867) (72.3%) (*P*=0.235). Additionally, married HCWs (n=964) (75.8%) and HCWs with a belief toward the efficiency of the vaccine (n=1,106) (86.1%) were more likely to accept the vaccine (*P*<0.001 for both).

Declared source of information about COVID-19 vaccines significantly influenced HCWs’ decision to get immunized. When taken individually, HCWs who used scientific journals and WHO announcements as sources of information about COVID-19 vaccines were more inclined to accept COVID-19 vaccines compared to those who do not (n=899 (79%) versus n=410 (62.8%) and n=959 (74.9%) versus n=351 (68.6%), respectively) (*P*<0.001 and *P*=0.006, respectively). On the contrary, newscasts, social media, and announcements by the Ministry of Health as information sources seemed to have a negative effect on HCWs’ decision to get vaccinated against COVID-19 (n=762 (70%) versus n=548 (78%), n=811 (71%) versus n=499 (77.97%), and n=1,004 (71.46%) versus n=306 (79.07%), respectively) (*P*<0.001, *P*<0.001, and *P*=0.003, respectively). The perceived risk of having severe COVID-19 was statistically higher in the vaccine acceptors (3.32) compared to that in COVID-19 vaccine refusers (3.01) (*P*<0.001).

Elimination of COVID-19 vaccine hesitancy

Those who would refuse to be inoculated with the COVID-19 vaccine expressed that they would possibly change their decision to refuse vaccination on the condition that people around them (n=514) (29.2%), physicians (n=504) (28.6%), or ministers and high officials (n=408) (23.2%) get vaccinated. Vaccine refusal would persist in 334 (19%) HCWs despite any role modeling behavior.

Attitudes on voluntary and mandatory vaccination against COVID-19

Slightly more than half (n=1,052) (58.5%) of the HCWs thought vaccination against COVID-19 should not be mandatory.

Preference for COVID-19 vaccines

The Pfizer-BioNTech vaccine was the most preferred COVID-19 vaccine (n=665) (37.3%). Sinovac vaccine CoronaVac®, with which large masses have been inoculated in the first several months of the vaccination process in Turkey, came in second, followed by Oxford/AstraZeneca, Sinopharm, Moderna, and others (Table 1).

**Table 1:** Healthcare workers’ preference of COVID-19 vaccines

Vaccine brand/manufacturer	Number of healthcare workers (%)
Pfizer-BioNTech	665 (36.9)
Sinovac (CoronaVac^®^)	417 (23.4)
Oxford/AstraZeneca	55 (3.1)
Sinopharm	36 (2)
Moderna	31 (1.7)
CanSino	17 (1)
Other	19 (1.1)
“Whichever”	247 (13.8)
“None”	298 (16.7)
Total	1,785 (100)

The following reasons were mentioned by the HCWs for preferring the Pfizer-BioNTech vaccine: trustworthiness of the manufacturing country of origin (Germany), trustworthiness of the manufacturer company, country of origin of its developers (Turkey), its uniqueness in being the first COVID-19 vaccine to receive emergency validation from the WHO, and its non-Indian, non-Russian, and non-Chinese origin.

The main reasons for HCWs’ preference of CoronaVac® were as follows: vast amount of experience on inactivated vaccines, high safety profile of inactivated vaccines, speculated relative safety for autoimmune phenomena, and expectation to be less affected by viral genetic mutations.

Parental willingness to vaccinate children against COVID-19

A total of 199 HCWs (15.6% of the responders of the relevant question) would never give consent to the inoculation of their children with a COVID-19 vaccine even if the safety and effectiveness of these vaccines in children are more firmly established in the future. There were 407 (31.9%) HCWs experiencing hesitancy in their children’s vaccination. Six hundred seventy-two (52.5%) HCWs declared their acceptance to get their children immunized with a COVID-19 vaccine. A marked increase in parental willingness on their children’s vaccination against COVID-19 was noted in HCWs who are 51 years of age and over (*P*<0.001).

## Discussion

To the best of our knowledge, this is the first and only national study reporting HCWs’ opinion on COVID-19 vaccines in Turkey. In congruence with our study population, nearly a fifth of the HCWs had a chronic medical condition in a French study, and contrary to our findings and to our surprise, having a chronic disease did not seem to affect the COVID-19 vaccine acceptance of French HCWs [7]⁠.

The significantly higher tendency of married individuals toward accepting COVID-19 vaccines has been established in previous studies, as is true for people with a strong belief in the efficiency of these vaccines [8,9]⁠. We found similar results in our study.

The majority of HCWs were against mandatory vaccination, which was in parallel with the Turkish government’s policy of convincing the public about the necessity of vaccination against SARS-CoV-2 for making mass vaccination possible. According to the results of a German study, 70% of the participants from the general population would voluntarily get vaccinated, and half of the participants were in favor of mandatory vaccination [10]⁠.

Of the Turkish HCWs, 41% perceived the risk of having severe COVID-19 as moderate, which was similar and comparable with the results of an Italian study, where the participants, 15.80% having been infected with SARS-CoV-2, declared that they were worried about COVID-19 with a median score of 3 (IQR: 2-3 out of 4). The perceived risk of having severe COVID-19 for nurses, observed significantly higher than in other aforementioned professional categories in our study, is understandable and consistent with nurses’ relatively long bedside activity and direct contact with COVID-19 patients, as similarly noted in an Italian study [11]⁠.

A commercial research company revealed that the COVID-19 vaccine refusal in Turkey was as high as 48.5% [12]⁠. In another poll by another research company, the proportion of people who are indecisive on being vaccinated, those who most probably will refuse vaccination, and strict refusers amounted to 30%, 10%, and 8% of the population, respectively [13]⁠. In our study, the rate of COVID-19 vaccine acceptors in Turkish HCWs was much more than that found in the Turkish general population.

The willingness of Turkish HCWs to be immunized against COVID-19 was much higher (73%) than those of Congolese HCWs (28%) and at comparable levels with those of French (78%) HCWs and those living in the United States (93%) [14-16]⁠. For further comparison, in a study conducted in the francophone regions of Belgium and Canada, COVID-19 vaccine acceptance was 76% and 69%, respectively [17]⁠.

We learn from two studies conducted among nurses in Hong Kong that 40%-63% of nurses had the intention to accept COVID-19 vaccination, which is much less than Turkish nurses’ willingness to get vaccinated (58%) [9,18]⁠. In contrast to Maltese HCWs, intention to get vaccinated against COVID-19 did not significantly differ between genders in our study, but the professional category that was most likely to get vaccinated was physicians, as was the case in the Maltese study [19]⁠. Similar differences in COVID-19 vaccine acceptance rates between various professional categories were previously observed in Israel and France [7,19]⁠.

Role modeling can be defined as direct or indirect interactions with significant others that potentially influence an individual’s beliefs, attitudes, and behaviors through the process of modeling; thus, it is critical to step up immunization in a country [20]. If HCWs, as role models for the public, are not vaccinated, then individuals who discover this hesitancy would have a tendency to avoid vaccination, as reported in a Turkish qualitative study [21]⁠. What is more disturbing is that the vaccine refusal rate in HCWs (4.2%) was very similar to that in the general population (5%) in Turkey [13]⁠.

For the majority (81%) of vaccine refusers in our study, acceptance of COVID-19 vaccines would be possible on the condition that physicians or ministers and high officials get vaccinated. This is a known situation from previous research that willingness to receive the vaccine is primarily influenced by powerful others’ actions [22]⁠. This not only emphasizes the importance of role modeling in widening vaccine acceptance and diminishing vaccine refusal but also means that HCWs as role models for vaccination actually need their own role models as well.

When the currently available COVID-19 vaccines were listed in the order of preference, HCWs and the Turkish public were in perfect harmony in choosing the top three (Pfizer-BioNTech, Sinovac, and Moderna vaccines). Of the Turkish population, 27% did not trust any of the vaccines, while this mistrust was less prevalent (16.7%) among HCWs [23]⁠. Libyan HCWs did not agree with their Turkish counterparts for the Pfizer-BioNTech vaccine was their last choice and their mistrust toward any COVID-19 vaccine (47.1%) was higher than that of Turkish HCWs [24]⁠.

Turkish HCWs were more concerned about the possible adverse effects of COVID-19 vaccines (56.2% for short-term adverse effects and 79.7% for long-term adverse effects) than the Turkish public (58%) in general. Of the HCWs, 28% thought the COVID-19 vaccine they will receive would not be protective enough, a pessimism much more prevalent than that observed in the public (8%) [13]⁠.

In a qualitative study conducted in Croatia, France, Greece, and Romania, the mistrust in COVID-19 vaccines was directed mostly toward health authorities or pharmaceutical companies, but not toward certain countries, which was the case in our study [25]⁠. In the past, there was a prevailing public opinion in Turkey that Chinese merchandise was shoddy, partly because of its low price and low quality. Although this misconception has changed over time, responses in our open-ended questions show, although qualitatively, that some people still think in the same way, explaining their standpoint for COVID-19 vaccines. Another explanation for the distrust among Turkish HCWs to COVID-19 vaccination could well be the unconvincing, conflicting, or vague statements by health authorities and academicians.

In a study conducted in Israel, parental willingness to vaccinate children against COVID-19 was 60% for physicians, 55% for nurses, and 70% for the general population [2,19]⁠. In a Canadian study of 380 parents, 61% responded that they were very likely to have their child vaccinated against COVID-19, and 25% said that they were somewhat likely [26]⁠. In Germany, COVID-19 vaccine hesitancy was considerable among parents both personally (42%) and filially (49%) [27]⁠.

A more recent Turkish study confirmed this low willingness for parental vaccination in the country. While 59.9% of parents were willing to receive a COVID-19 vaccine themselves, only 36.3% of them agreed to have their children vaccinated. Parental vaccine acceptance was significantly higher among HCWs (47.2%). The researchers of this study found age as a factor favoring parental vaccine acceptance as we did [28]⁠.

Study strengths and limitations

In terms of strength, this is the first study in Turkey that we know of to reveal HCWs’ opinions about COVID-19 vaccines and their personal and parental intentions of COVID-19 immunization. The number of participants well exceeded the calculated sample size. Another strength may be the guaranteed anonymity of responses with “no name required” via an online survey platform instead of a face-to-face or telephone interview. Our study also provides useful information for national and foreign health policymakers on the current status of and the factors related to HCWs’ personal and parental hesitancy on COVID-19 vaccination of children less than 12 years of age.

One of the limitations and biases of this study may involve its cross-sectional nature, where the answers were self-reported. Professional categories within HCWs may be overrepresented or underrepresented proportionally. Another limitation may be the lack of follow-up evaluation, which is especially necessary to determine the realization of the reported vaccine rejection (26.9%) among HCWs. Other limitations are related to the use of an unvalidated questionnaire and a nonrepresentative sample of the overall Turkish HCWs. Likewise, we did not collect data regarding the region of residence; therefore, regional variations may exist. Additionally, as a questionnaire-based study, it was subject to social desirability bias, i.e., participants may have given answers to meet the expectations of their familial or professional circles.

## Conclusions

Our findings highlight the unacceptably high prevalence of rejection and hesitancy among HCWs regarding personal and parental vaccination against SARS-CoV-2. Anti-vaccine attitudes among HCWs jeopardize public acceptance of COVID-19 vaccines since HCWs are known to be critical role models for stepping up immunization in a country. A low acceptance rate of COVID-19 vaccines within the healthcare community may lead to the low acceptance of these vaccines within the public in general, thus paving the way to delayed herd immunity at the expense of high morbidity, mortality, and subsequent financial burden to the national economy. This ominous “domino effect” may come true unless necessary measures, such as persuasion of HCWs on personal and parental COVID-19 vaccination by providing information about the benefits of these vaccines, are taken as soon as possible.
